# Supplementary figures and images for: Modeling of H5N1 influenza virus kinetics during dairy cattle infection suggests the timing of infectiousness
Source: PLoS Biol. 2026 Jan 5;24(1):e3003586. doi: 10.1371/journal.pbio.3003586 (PMC12782433; doi:10.1371/journal.pbio.3003586)

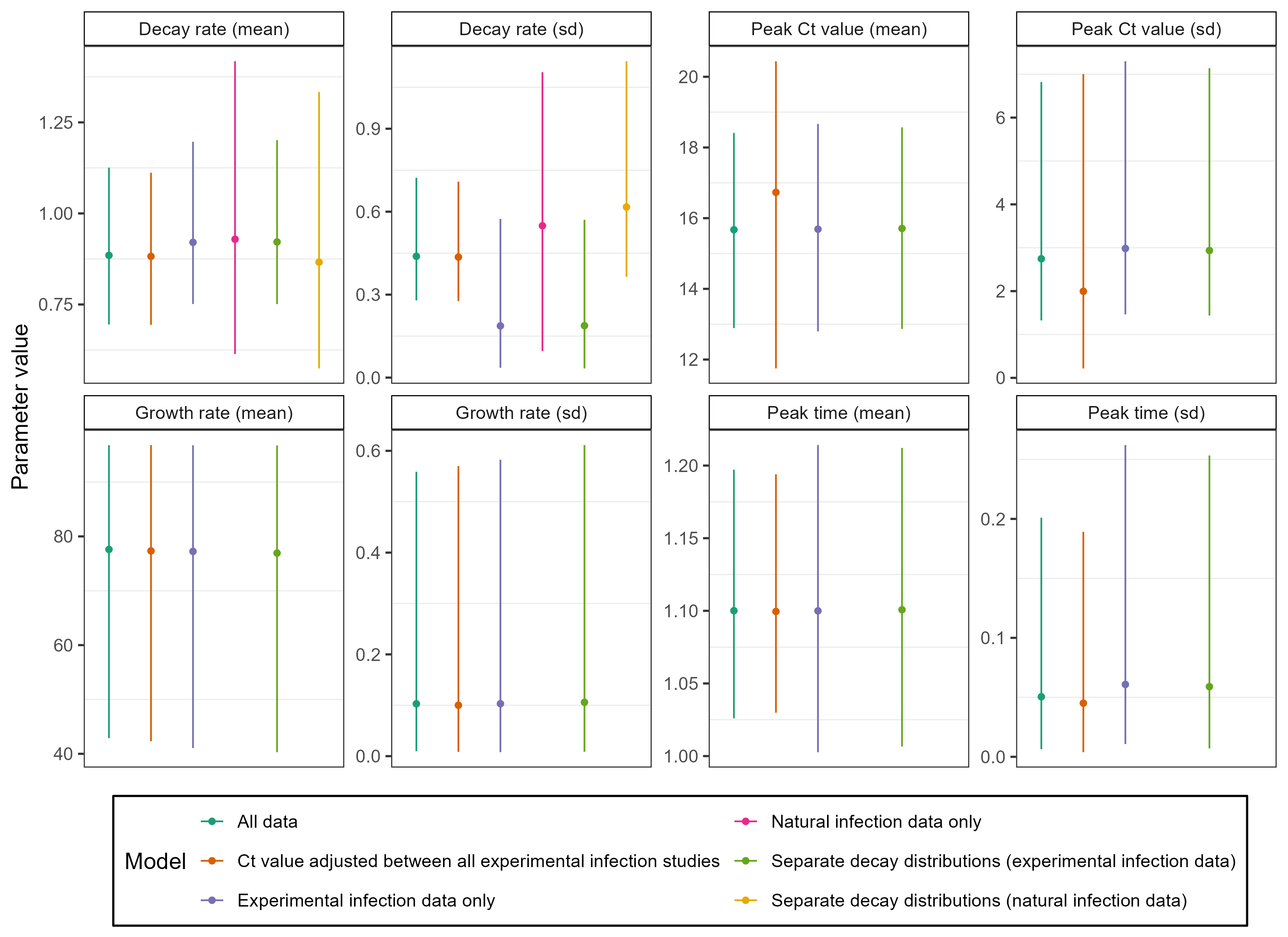

Supplement: S1 Fig — Median (point) and 95% credible intervals (lines) for posterior parameter estimates for the Ct value trajectory model using different subsets of data. Models include: fitting to all data, the main model used for analysis (dark green); fitting to all data but including an additional parameter that adjusts the modeled Ct values between the data from the two different experimental infection studies (orange); fitting only to the data for experimentally infected cattle (purple); fitting only to the data for naturally infected cattle (pink); and fitting to all data, but allowing separate distributions for the viral decay rate between experimentally and naturally infected cattle (green for experimentally infected cattle, yellow for naturally infected cattle). Note that because naturally infected cattle are generally detected post peak the model fit to data from naturally infected cattle only estimated the viral decay rate distribution. The data underlying this figure can be found in S1 Data and at http://dx.doi.org/10.5281/zenodo.17604863. (TIFF) [file pbio.3003586.s001.tiff]

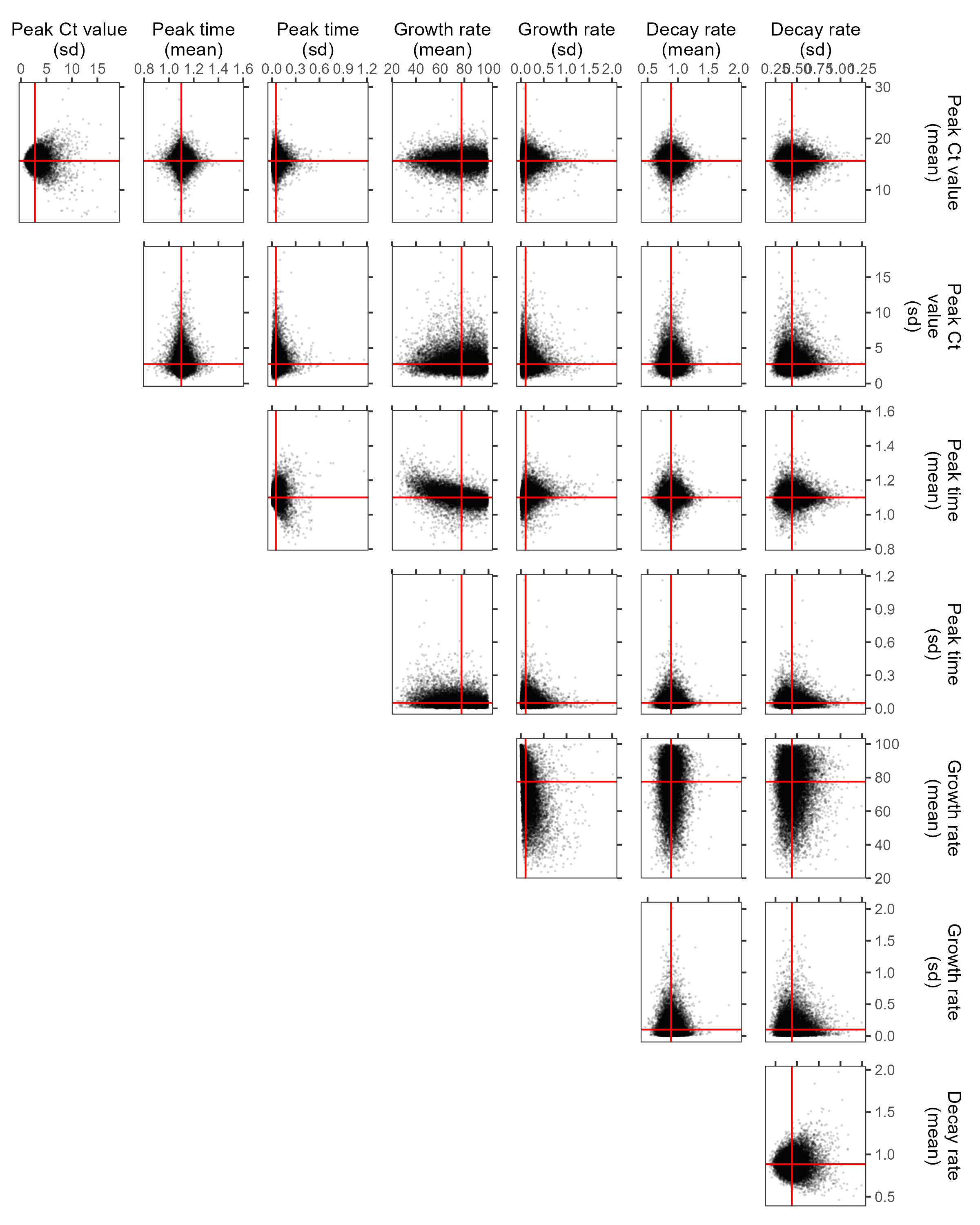

Supplement: S2 Fig — Posterior samples (black points) from the Ct value model for all population parameters (means and standard deviation). The distribution of posterior samples is shown for each pair of population parameters. Also shown is the median of the posterior distribution for each individual parameter (red lines). These median parameter values are also shown in SFig.1 and were used in Fig.2a to plot the 95% interval of the estimated population distribution of Ct value trajectories (using median parameter estimates). The data underlying this figure can be found in S1 Data and at http://dx.doi.org/10.5281/zenodo.17604863. (TIFF) [file pbio.3003586.s002.tiff]

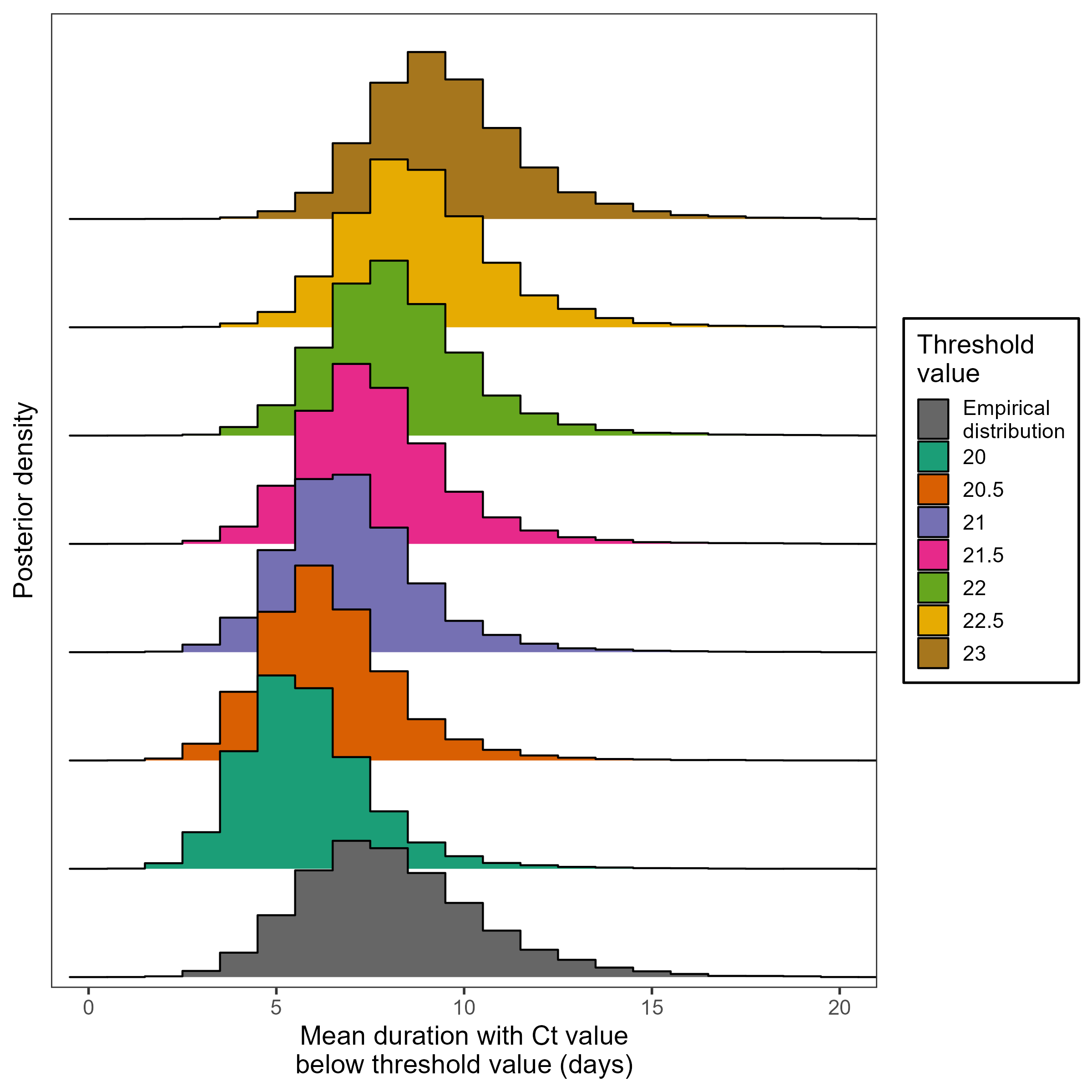

Supplement: S3 Fig — The posterior distributions for estimates of the mean duration for which cattle’s Ct value remains below a threshold value. Shown are the posterior distributions for different choices of the threshold value (colors). Also shown is the posterior distribution using the empirical distribution of the threshold value (as estimated using the model linking Ct value and the log-titer of infectious virus, 2 parameter). Ct values above the estimated threshold value represent little-to-no infectious virus and so are unlikely to be infectious. This posterior distribution thus represents our posterior distribution for the duration of infectiousness. The data underlying this figure can be found in S1 Data and at http://dx.doi.org/10.5281/zenodo.17604863. (TIFF) [file pbio.3003586.s003.tiff]

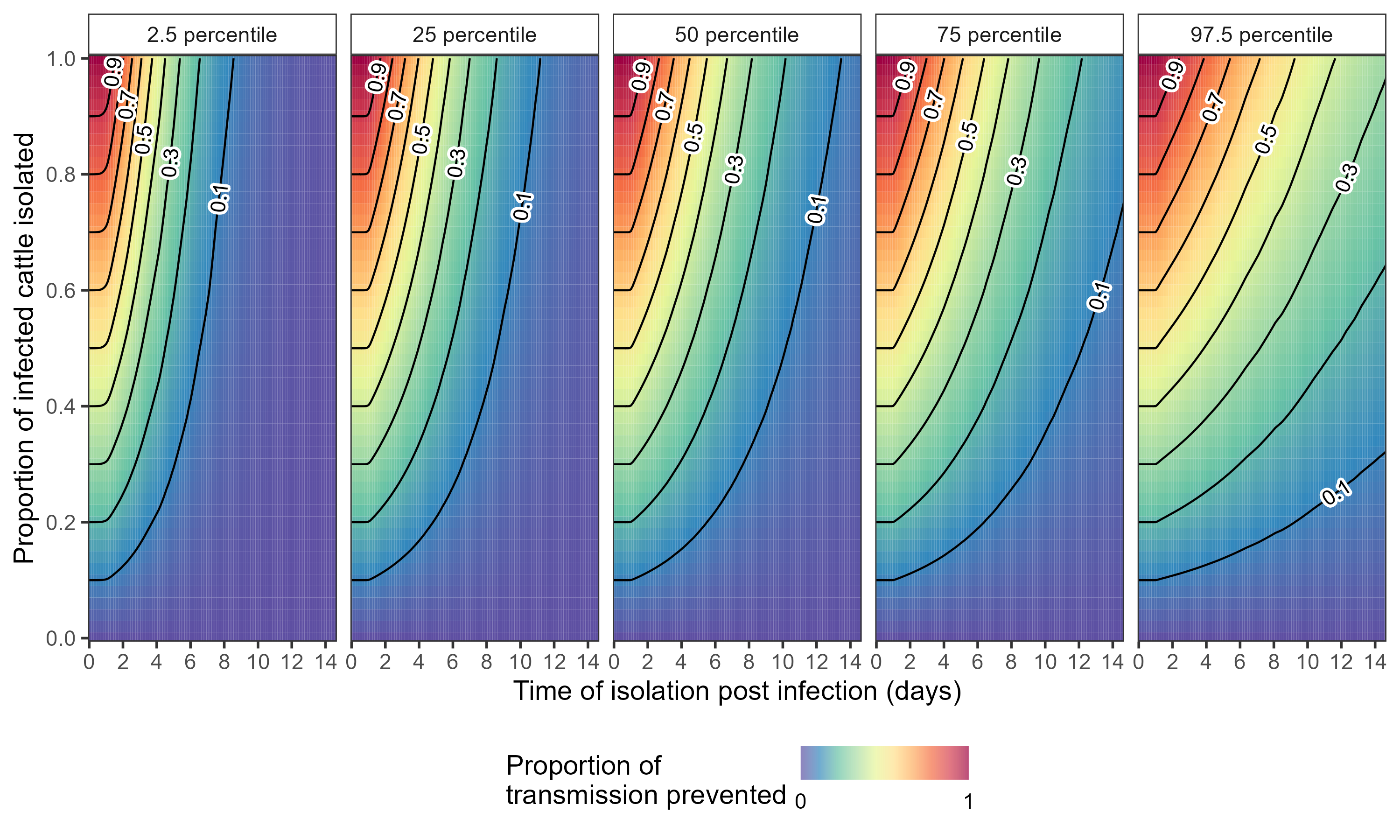

Supplement: S4 Fig — The proportion of transmission events prevented (color) as a function of: the time post infection at which infected cattle are isolated (x-axis); and the proportion of infected cattle that are isolated (y-axis). The proportion of transmission events prevented is calculated across the entire posterior distribution for both models; plotted is the median value (50 percentile panel), and the central 50% (25–75 percentile panels) and 95% (2.5–97.5 percentile panels) credible intervals. Contour lines (proportion of transmission prevented: 0.1–0.9 in intervals of 0.1) connect points on each panel with the same value (black lines). The data underlying this figure can be found in S1 Data and at http://dx.doi.org/10.5281/zenodo.17604863. (TIFF) [file pbio.3003586.s004.tiff]

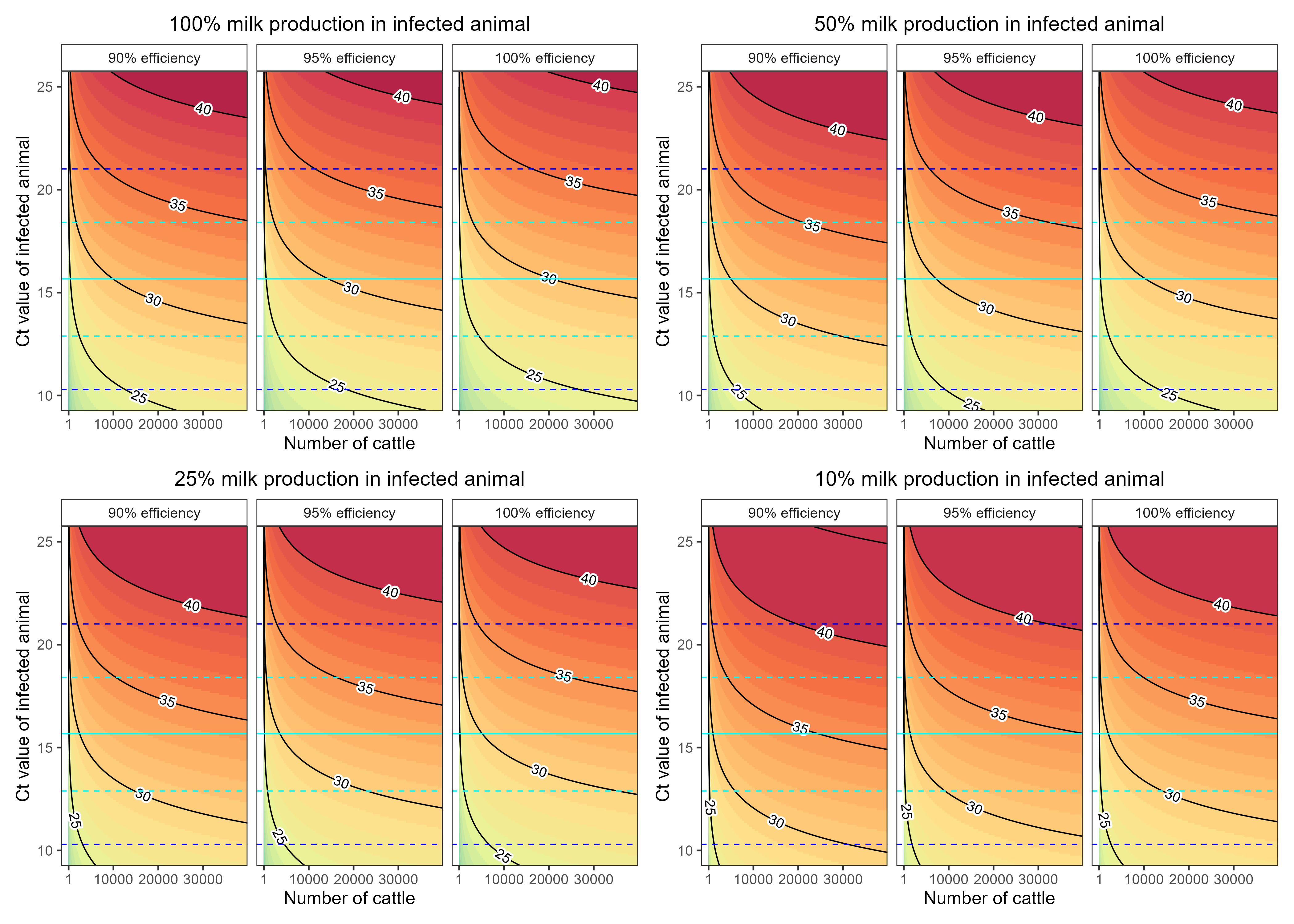

Supplement: S5 Fig — Expected Ct value of a pooled milk sample (color) as a function of: the Ct value of the infected animal (y-axis, one infected animal contributing to the pooled sample); the total number of cattle contributing to the sample (x-axis); the efficiency of the rt-PCR test used (panels); and the milk output of the infected animal relative to uninfected animals. Note that an rt-PCR test of 100% efficiency doubles the viral concentration with each round of amplification (Ct value increasing by 1). Contour lines (Ct values: 25, 30, 35, 40) connect points on the graph with the same value (labeled black lines). The median (solid line) and 95% credible interval (dashed lines) for the population mean minimum Ct value (P) for a single infected animal is highlighted (cyan). The 95% credible interval (dashed lines) for the population distribution of minimum Ct values using mean population parameters, N(P, P), for a single infected animal is also highlighted (blue). The data underlying this figure can be found in S2 Data. (TIFF) [file pbio.3003586.s005.tiff]
