# Supplementary material for: Modeling of H5N1 influenza virus kinetics during dairy cattle infection suggests the timing of infectiousness
Source: PLoS Biol. 2026 Jan 5;24(1):e3003586. doi: 10.1371/journal.pbio.3003586 (PMC12782433; doi:10.1371/journal.pbio.3003586)
Supplement: Attachment — Submitted filename: Response to reviewers.pdf [file pbio.3003586.s011.pdf]

Dear Editors,

PBIOLOGY-D-25-01949R1

Thank you for your email on 25 August 2025 and your invitation to revise and re-submit our short report entitled “Viral kinetics of H5N1 infections in dairy cattle" for further consideration by PLOS Biology.

We thank the editor and reviewers for their comments.

We found the comments and suggestions from each reviewer to be very helpful and have made a number of revisions to the manuscript. In particular, we have incorporated data from Facciuolo et al. Nature Microbiology 2025 into our analyses (two experimentally infected cattle with longitudinal data up to 28 days post infection). Additionally, we have: performed sensitivity analysis to demonstrate the maintained value of pooled milk sample testing when milk output of infected animals is reduced by as much as 90%; and tempered our conclusions by communicating additional complexities that our models can not account for even when combining much of the limited published data. We provide a point-by-point response to the reviewer comments in our attached response letter. Each comment is listed in black, and our responses are in red.

We believe that the revised manuscript has been improved. I would be happy to answer any further questions that you or the reviewers might have and look forward to hearing your decision.

Yours sincerely,

Dr Oliver Eales

Reviewer #1:

Summary

This manuscript by Eales et al. presents a timely modeling effort to estimate viral kinetics, infectiousness, and testing sensitivity for H5N1 virus in dairy cattle. The topic is relevant given the ongoing spread of H5N1 in livestock and the urgent need for evidence-based surveillance and control strategies. The authors leverage limited available data to develop their models and highlight the importance of national surveillance efforts. However, several key assumptions warrant further clarification or tempering of conclusions. Additionally, emerging experimental data challenge aspects of the model outputs, raising questions about the robustness and biological relevance of some inferences.

We thank the reviewer for their thorough review and helpful comments. We have tempered our conclusions throughout the manuscript (please see edits in tracked changes) and have extended the limitations section.

Major Comments

1. The authors model Ct value trajectories in milk based on published data from experimentally and naturally infected cattle. However, the infectious inoculum used in experimental infections likely differs from natural exposures, both in dose and route. Prior studies (e.g., Lee et al., https://www.researchsquare.com/article/rs-6900680/v1) have demonstrated that viral kinetics, including the timing and magnitude of peak viral load, can be influenced by infectious dose. Can the authors address how such differences might influence the trajectory parameters in their model?

We have included in the limitations section a discussion on the representativeness of the experimentally infected cattle:
While our analysis found no evidence for a difference in the viral decay rates between experimentally and naturally infected cattle, the only longitudinal data that measured Ct values over the entire course of infection was from experimentally infected cattle; viral kinetics of H5N1 infection in naturally infected cattle may differ. For example, naturally infected cattle may have been initially exposed to a lower volume of infectious virus than experimentally infected cattle (a high volume of infectious virus is often used during experimental inoculation to ensure infection), which could result in a longer time until the peak Ct value is reached [14]. **Indeed, one study that inoculated a cow with a significantly lower volume of infectious virus (than the experimental infection studies whose data were analysed in this paper) and found a longer duration to peak in viral loads [15]**." (Lee et al)

We additionally discuss how such difference might influence our inferred outcomes: "Differences in the time to minimum Ct value would alter the period for which **we estimate that** cattle remain infectious **(assuming the same rate of viral decay)** and thus alter our estimates of the effectiveness of control measures such as case isolation. **For example, if the time to peak viral load (minimum Ct value) was longer in naturally infected cattle (e.g.**

as reported in Lee et al) then we might expect a longer delay between infection and an animal becoming infectious, increasing the effectiveness of case isolation (more cattle isolated earlier in their infectious period)."

2. A recent study by Facciuolo et al. (PMID: 40247094) also characterized viral growth kinetics in experimentally infected dairy cattle using milk samples. Given the limited sample size in the present study (Fig. 1A), the authors should consider incorporating or comparing their estimates with those reported by Facciuolo et al. to strengthen the robustness of their presented dataset.

As the reviewer suggested, we have incorporated data from the recent study Facciuolo et al. into our analyses. We have updated our estimates throughout accordingly, and we have detailed the additional data in the Methods section. Our estimates are still broadly the same as our previous analysis (not including data from Facciuolo et al), with credible intervals overlapping significantly.

3. In Figure 3, the authors model the expected Ct value of pooled milk samples and conclude that a single index case could be detected in large dairy herds (30,000-40,000 cattle) through milk vat testing. However, it is unclear how the model accounts for milk volume contributions from infected versus uninfected animals. Several cited studies report that clinically infected cattle exhibit marked reductions in milk production—particularly prior to or near peak viral loads. If a symptomatic cow contributes little or no milk to the bulk tank at the time of peak viral shedding, this would substantially reduce the effective viral concentration in pooled milk. Can the authors clarify whether this was incorporated into their calculations, or whether assumptions of uniform milk contribution may overestimate detection sensitivity?

We have now specified that uniform milk contributions from infected and uninfected animals were assumed, and that this may lead to an overestimate in detection sensitivity:
"For a milk sample from one animal with a Ct value of 15.7 (the population mean peak Ct value), **assuming no reduction in milk output**, …"
"**These estimates assume that an infected animal produces the same milk output as healthy cattle and thus is likely an overestimate of the sensitivity of pooled testing.**"
We have also added a sensitivity analysis (supplementary figure) looking at the expected Ct value of pooled milk samples if milk production of an infected animal was 50%, 25%, or 10% that of an uninfected animal. We have added to the results text:
"**However, even when assuming a reduced milk output for infected animals as low as 10% of an uninfected animal (SFig.5), we found that a typical Ct value (population mean peak Ct value) was likely to be within realistic limits of detection.**"

4. The data presented in Figure 2B suggest a relationship between Ct value and infectious virus titer, with a rapid decline in recoverable infectious virus centered around a Ct value of 21.5. However, this finding, based on a single study, may overstate the existence of a strict infectivity threshold. Influenza viruses—including H5N1—have been isolated from primary samples with Ct values above this range. Given the cross-sectional nature of the data and the lack of longitudinal measurements from individual animals, the model may not

adequately reflect the true variability in infectious virus recovery. For example, in a report by Halwe et al., a relationship between Ct value (Fig. 3C) and infectious titer (Fig. 4A) demonstrates that Ct values >21 can still yield infectious virus. The authors should consider either supporting this relationship with additional experimental data or tempering their conclusions accordingly.

We agree with the reviewer and have tempered our conclusions accordingly.
We have extended our paragraph in the limitations:
"We had limited longitudinal data describing the relationship between measured Ct values and the log-titre of infectious virus, and so could not fit a hierarchical model that allows for variation in the relationship between individuals in the population. **Hence our model assumed a population-level relationship between Ct values and the log-titre of infectious virus; as such the 'critical threshold' Ct value that we estimated represents a population average (for this specific sample of cattle, which may not be representative of the entire dairy cattle population). There may be individual-level variation in this relationship, with some cattle still yielding infectious virus when their Ct value has passed above this population-level threshold value (e.g. Halwe et al.).** Individual-level variation could influence estimates of the distributions of the duration and timing of infectiousness."

5. The infectiousness model presented in Figure 2C would benefit from additional context based on recent experimental findings. Lee et al. (https://www.researchsquare.com/article/rs-6900680/v1) showed that as little as 10 TCID50 of virus was sufficient to establish infection in the bovine mammary gland, suggesting that infectious dose thresholds may be lower than assumed in the model. This raises the possibility that animals with Ct values above the modeled "critical threshold" could still contribute to transmission.

Thank you for referring us to the work of Lee et al. We have more thoroughly discussed limitations in our estimates of duration of infectiousness in the Limitations section. Please see the tracked changes version for the full discussion. However, we include below the new section highlighting potentially lower infectious dose thresholds as reported by Lee et al (reference #16):
"Low but non zero infectious viral load later in the infection may skew the infectiousness profile to later times; a recent study showed that as little as 10 TCID50 /ml of virus (below or at the limit of detection of the data used in our analysis) was sufficient to establish infection in the bovine mammary gland [16]."

6. The estimate that 95% of cattle are no longer infectious by 16.6 days post-infection does not appear to account for host immune responses. While viral RNA may persist in milk for some time (>10 days), the presence of neutralizing antibodies—especially in recovered animals—is likely to substantially reduce or eliminate the potential for onward transmission. For example, Halwe et al. report difficulty isolating virus beyond 9 days post-infection, despite RNA-positive milk, likely due to neutralizing antibodies. The 16.6-day estimate may overstate infectious potential in natural settings. Incorporating immune dynamics, or at least acknowledging this limitation, would strengthen the interpretation of the infectious period.

We have more thoroughly discussed limitations in our estimates of duration of infectiousness in the Discussion section. Please see the tracked changes version for the full discussion. We have included the additional paragraph below (that specifically discusses antibody response/host immunity):
“Additionally, our modelling assumption of a constant relationship between Ct value and the log-titre of infectious virus, does not consider other possible non-linear interactions with the host immune response. Halwe et al. (Halwe et al. 2024) found that antibody levels increased from ~7–11 days post infection in two experimentally infected cattle, with neutralising antibodies detected in milk samples from nine days post infection. It stands to reason that the presence of neutralizing antibodies is likely to substantially reduce or eliminate the potential for onward transmission. The antibody response will (we expect) be induced by the presence of infectious virus; thus quantifying a relationship between Ct value and the log-titre of infectious virus (as we have done) can implicitly account for some of the effects of underlying (causally related) antibody dynamics (that reduce the infectious viral load). However, our statistical model assumed (by necessity) a simple relationship between Ct value and the log-titre of infectious virus, which may not be able to quantify a more complex relationship between Ct value, log-titre of infectious virus, and antibody response. Ideally, more longitudinal studies that measure Ct value trajectories would also concurrently collect data on the log-titre of infectious virus and antibody levels. Models may then be developed that quantify the (potentially non-linear) relationships between these three quantities, while accounting for variation between individual cattle.”

Minor Comments
Please double-check citation numbering. Caserta et al. is labeled as "[4]" in the Methods section but appears as "[3]" in the reference list.

Thank you, we have now fixed the numbering of the references.

Reviewer #2:

In the manuscript by Eales et al, the authors seek to quantify epidemiological parameters associated with H5N1 outbreaks on dairy farms through analysis of published data from 3 studies (2 experimentally infected and 1 naturally infected). The authors use published data to suggest that infectious virus would only be observed in milk samples with a Ct value less than or equal to 21.5 and that the average duration for shedding of infectious virus of 6.2 days post infection. The authors also model the theoretical sensitivity of qPCR to detect a single H5N1 positive animal and conclude that this is feasible within 30,000 cows if a single cow has a CT value <25. While the conclusions are reasonable based on the data used to generate the model, they are not representative of the complexity of the ongoing H5N1 outbreak on dairy cattle. Thus, the utility of the analysis is significantly limited.

We thank the reviewer for their thorough review and helpful comments. We have tempered our conclusions throughout the manuscript (please see edits in tracked changes) and have extended the limitations section.

Major comments:
1. The authors have a limited number of cows in the analysis - 5 experimentally infected cows and 12 naturally infected cows (from a single farm). Based on the graphs presented in the manuscript the kinetics of shedding seem heterogenous in the naturally infected animals compared to the experimentally infected animals. It is unclear how similar the kinetics are across the different infection conditions or across animals in the naturally infected group. Addition of more animals could help refine the different infection kinetics that are present during an outbreak. Additional publications to include: (Facciuolo et al, Nature Microbiology; Faccin, F.C. et al. doi.org/10.21203/rs.3.rs-6915991/v1, Lee, C. et al. doi.org/10.21203/rs.3.rs-6900680/v1)

As the reviewer suggested, we have incorporated additional data into our analyses. We have included data from an additional two experimentally infected cattle from Facciuolo et al.2025 Nature Microbiology. We have updated our estimates throughout accordingly, and we have detailed the additional data in the Methods section. Our estimates are still broadly the same as our previous analysis (not including the data from Facciuolo et al), with credible intervals overlapping significantly. We were unable to include additional data from Faccin et al. as standard curves were requested from the authors (early-October) but not made available to us within the timeframe allowed for manuscript resubmission (i.e. we are yet to hear back from them on this request). We were not able to include data from Lee et al. as the data were not available. We have extended our limitations section to discuss potential heterogeneity in infection kinetics across different infection conditions and how these might impact our findings:
"There may be issues with the representativeness of the data, given the small number of animals sampled and the lack of data from naturally infected animals. **While our analysis found no evidence for a difference in the viral decay rates between experimentally and naturally infected cattle,** the only longitudinal data that measured Ct values over the entire course of infection was from experimentally infected cattle; viral kinetics of H5N1 infection in naturally infected cattle may differ. For example, naturally infected cattle may have been

initially exposed to a lower volume of infectious virus than experimentally infected cattle (a high volume of infectious virus is often used during experimental inoculation to ensure infection), which could result in a longer time until the minimum Ct value is reached [15]. Indeed, **one study that inoculated a single cow with a significantly lower volume of infectious virus (than the experimental infection studies whose data were analysed in this paper) and found a longer duration to peak in viral loads** [16]. Differences in the time to minimum Ct value would alter the period for which **we estimate that** cattle remain infectious **(assuming the same rate of viral decay)** and thus alter our estimates of the effectiveness of control measures such as case isolation. **For example, if the time to peak viral load (minimum Ct value) was longer in naturally infected cattle (e.g. as reported in Lee et al (Lee et al. 2025)) then we might expect a longer delay between infection and an animal becoming infectious, increasing the effectiveness of case isolation (more cattle isolated earlier in their infectious period)**. Additionally, the data we had from naturally infected cattle were identified through early outbreak investigations, which may have been preferentially biased towards more symptomatic or severe cases. This underscores the need for more detailed studies aimed at capturing the full course of infection in naturally infected cattle, with unbiased selection of cattle — longitudinal sampling of randomly selected cattle on a dairy farm during an outbreak could provide such data."

2. Because the analyses in this paper are almost all based on the idea that Ct value can be linked to infectious virus, it is critical to note properties that can influenza infectious titer in milk. Specifically, this manuscript makes no mention of induction of host immune responses and the role they may play in the duration of infectiousness. In each of the 2 controlled infection model studies used for this analysis, antibody titers are tracked over time, thus it is known when post-infection antibody levels started to rise. A raise in antibodies within the milk as infectivity drops is an important consideration for CT to infectious titer analysis. In Baker et al, infectious virus is isolated at 12dpi but not after and that cow had a virus neutralization titer of 160 by that day; in each cow, neutralizing antibody titers had started to rise by 9-10dpi, respectively. In Halwe et al antibody levels rise ~ 7-10 days post inoculation. Since these datasets are used in these analysis reported, it is an oversight not to mention that these antibody responses likely relate to lack of recovery of infectious virus.

We thank the reviewer for highlighting this oversight. We have more thoroughly discussed limitations in our estimates of duration of infectiousness in the Discussion section. Please see the tracked changes version for the full discussion. We have also included the additional paragraph below (that specifically discusses antibody response/host immunity):
"Additionally, our modelling assumption of a constant relationship between Ct value and the log-titre of infectious virus, does not consider other possible non-linear interactions with the host immune response. Halwe et al. (Halwe et al. 2024) found that antibody levels increased from ~7–11 days post infection in two experimentally infected cattle, with neutralising antibodies detected in milk samples from 9 days post infection. The presence of neutralizing antibodies is likely to substantially reduce or eliminate the potential for onward transmission. The antibody response will (we expect) be induced by the presence of infectious virus; thus quantifying a relationship between Ct value and the log-titre of infectious virus can implicitly account for some of the effects of underlying (causally related) antibody dynamics (that reduce the infectious viral load). However, our statistical

model assumed (by necessity) a simple relationship between Ct value and the log-titre of infectious virus, which may not be able to quantify a more complex relationship between Ct value, log-titre of infectious virus, and antibody response. More longitudinal studies that measure Ct value trajectories should concurrently collect data on the log-titre of infectious virus and antibody levels. Models may then be developed that quantify the (potentially non-linear) relationships between these three quantities, while accounting for variation between individual cattle.”

Specific comments:

1. The manuscript title is misleading as there is no novel data on the viral shedding. Please revise to be more representative of the analysis conducted.

We do not think the title is misleading as we have quantified the ‘viral kinetics of H5N1 infections in dairy cattle’ using a statistical model. The title in no way suggests that data has been collected. However, we would be happy to change the title if the editor thinks it is necessary.

2. It might help the organization and flow of the manuscript if the 'Pooled testing of milk vats' section was moved to just before the 'Limitations' section. Currently, subpanels in the figures are discussed out of order which could be avoided by moving the 'pooled milk' section to the end. Rearranging this would also allow all analyses of samples from individual cows (Ct value trajectories, Ct x log titer, duration of infectiousness, and the discussion of isolation efficiency) to be discussed together, which seems like a more logical flow.

Thank you for the helpful suggestion. We have moved the “Pooled testing of milk vats” section to just before the limitations section as suggested. The figures are now referenced in order in the text.

3. Details are insufficient for the following areas and additional references are needed:
a. National Milk Testing Strategy (NMTS) - is wide spread use, strategy, limitations, and achievements.

We have included information on the NMTS:
“...recent implementation of the United States Department of Agriculture’s National Milk Testing Strategy [21] which includes: testing of samples from milk silos at national dairy processing facilities to determine the presence of H5N1 at the state level; and bulk tank sampling programs in (identified) affected states to identify dairy herds affected by H5N1. The testing strategy is designed to increase USDA’s and public health partners’ understanding of where the virus is present in the United States. It has been implemented in 46 states of the United States, allowing states and herds to be classified based on their H5N1 affected status, and hence interventions and resources to be better targeted [21].”

b. Impact of clinical vs subclinical cows is examined in Peña-Mosca et al, 2025, Nature Communications, and should be included and discussed.

Thank you for bringing our attention to this excellent paper. The results of this paper have improved our discussion of results on case isolation. We have included the following reference to Peña-Mosca et al in our discussion of clinical vs subclinical infection rate:
“A recent seroprevalence study, following an outbreak of H5N1 on a dairy farm, estimated that approximately 75% of infections were subclinical [13]; although this value will be sensitive to the definition of clinical infection used (i.e. how severe/identifiable the symptoms used to define a clinical case were)”
and:
“However, it will be difficult for high proportions of infections to be detected if a high fraction of infections are subclinical, as has recently been estimated [13], reducing the effectiveness of case isolation.”

Minor comments:
1. The authors need to carefully read over the manuscript.
a. Edit references to 'high' and/or 'low' Ct values as appropriate. In a couple places the manuscript reads 'high' Ct values when the context of the sentence leads me to think 'low' Ct values (high viral load) was intended, and vice versa. The abstract has a couple instances of this, as well as the penultimate sentence of Introduction.
b. Check for misspellings - one example: figure one: 'free' should be 'three'.

Thank you for raising this. We have now carefully reread the manuscript and fixed references to high/low Ct values. We have also corrected the highlighted misspelling and checked for others.

Reviewer #3:

The manuscript "Viral kinetics of H5N1 infections in dairy cattle" has been submitted to PLOS Biology by Eales, McCaw, and Shearer. In this paper, the authors assemble three sources of H5N1 kinetics data from dairy cattle. Two sources are experimental (mammary inoculation), and the other is observational. The paper proceeds logically by first developing a modeling approach that learns a posterior distribution of viral kinetics from all three data sources, considers the sensitivity of pooled testing, and then assembles the estimates required to evaluate the effectiveness of isolating infected cattle.

This is a valuable paper because it integrates data across studies to provide valuable estimates of the effectiveness of surveillance, the infectiousness/Ct relationship, and the potential effectiveness of isolation. The conclusions follow from the data, the limitations are appropriate, and the writing is clear. I support this paper's publication after minor revisions. Below, I provide some constructive suggestions.

We thank the reviewer for their thorough review and helpful comments.

In the analysis of Duration of Infectiousness, the authors provide estimates for the proportion of animals that do not become infectious, the upper 2.5% of infection durations, and the 95% credible interval for the duration of infectiousness. These values are estimated from a small sample size of mostly inoculated cattle, using distributions chosen for modeling convenience (but not because their tails are necessarily correctly specified). Therefore I recommend that these sorts of estimated tail values include an appropriate disclaimer to caution readers against overinterpretation.

We have now included a disclaimer immediately following our estimates:
"However, the estimated tail values of the infectiousness duration will be highly sensitive to the assumed distribution and so, these estimates should be interpreted with caution."

We have also more thoroughly discussed limitations in our estimates of duration of infectiousness in the Limitations section. Please see the tracked changes version for the full discussion. In particular, we include a paragraph on the assumption of a simple relationship between infectiousness and Ct value:
"Our model assumes a simple relationship between Ct value and infectiousness (specifically log-titre of infectious virus) — an animal with a Ct value below a threshold is infectious and above a threshold is not infectious. However, the infectiousness profile of an individual animal may vary more smoothly over time. The exact relationship would be difficult to quantify and would depend on: (1) the infectious viral load over time; and (2) the relationship between infectious viral load and infectiousness. Our estimate of the duration of infectiousness effectively assumes that: when the Ct value is below the threshold the infectious viral load is high (and constant) and the cow is infectious (with a fixed probability of infecting milking apparatus (given contact) per unit time); and when the Ct value is above the threshold, the infectious viral load tends to zero and the cow is not infectious. However, it is possible that the infectious viral load (and so infectiousness) changes more smoothly with Ct value (particularly above and below the threshold value). Higher Ct values

that we observe earlier in infection might indicate that cattle's infectiousness profile should be skewed to earlier times. Low but non zero infectious viral load later in the infection may skew the infectiousness profile to later times; a recent study showed that as little as 10 TCID50 /ml of virus (below, or at, the limit of detection of the data used in our analysis) was sufficient to establish infection in the bovine mammary gland [16].”

We also extended the first paragraph of the limitations to discuss how a delayed time to minimum Ct value might alter our estimates of the duration of infectiousness (and effectiveness of control measures):
“For example, naturally infected cattle may have been initially exposed to a lower volume of infectious virus than experimentally infected cattle (a high volume of infectious virus is often used during experimental inoculation to ensure infection), which could result in a longer time until the minimum Ct value is reached [15]. Indeed, **one study that inoculated a single cow with a significantly lower volume of infectious virus (than the experimental infection studies whose data were analysed in this paper) found a longer duration to peak in viral loads [16].** Differences in the time to minimum Ct value would alter the period for which we estimate cattle remain infectious **(assuming the samerate of viral decay)** and thus alter our estimates of the effectiveness of control measures such as case isolation**. For example, if the time to peak viral load (minimum Ct value) was longer in naturally infected cattle then we might expect there to be a longer delay between infection and an animal becoming infectious, this would increase the effectiveness of case isolation (more cattle isolated earlier in their infectious period). This is assuming that the viral decay rate following the peak is the same between naturally infected and experimentally infected cattle, and therefore that cattle remain infectious for a similar duration.** Our analysis found no evidence for a difference in the viral decay rates between experimentally and naturally infected cattle, but the sample size was small

The paper could also benefit from fleshing out the connection between pooled surveillance testing and cattle screening and isolation. Would it even be plausible to link surveillance and isolation-based interventions to suppress outbreaks quickly? For instance, the authors make the convincing case that weekly pooled testing is likely to detect outbreaks. And, they explore the proportion of transmission prevented by case isolation (particularly in Fig 4B). What happens when we put them together? What are the implications of the timing of detection via surveillance for the subsequent possible interventions? Is the first wave of secondary infections likely to be large because of a delay in detection, even if effective screening and isolation is possible? Does the delay between infection and symptoms present additional challenges to effective isolation? Rather than quantitative analyses that invoke additional assumptions these issues could be commented on, or discussed in the limitations -- which additional parameters would need to be nailed down before it would be possible to perform control analyses e.g. Kissler et al (PLOS Bio 2021) or Middleton et al (Sci Adv 2024)?

We have fleshed out the connection between pooled surveillance testing and case isolation as suggested adding text to the limitations section:

“We considered the effectiveness of case isolation and pooled testing of milk samples in isolation but did not consider their combined effect. When pooled testing is conducted regularly, outbreaks on dairy farms may be detected rapidly, allowing for the timely implementation of interventions. Case isolation is commonly used for preventing transmission of diseases afflicting dairy cattle (e.g. mastitis (Dairy Australia 2018)). We inferred the effect of case isolation on transmission, by estimating the proportion of transmission prevented as a function of the proportion of infections isolated and the time from infection to isolation. However, the effect of a transmission reducing intervention on the overall size of an outbreak will be highly dependent on the inherent transmissibility of the pathogen (i.e. the basic reproduction number, R0: the expected number of secondary infections arising from a primary infection in an otherwise susceptible population), and how soon after the onset of the outbreak the intervention is implemented. The earlier interventions are implemented, the smaller the outbreak. The higher the basic reproduction number (R0>>1), the smaller the impact of interventions. Note that if R0 is close to one, then only a small reduction in transmission would be required for an outbreak to be controlled (i.e. an immediate reduction in infections). However, this is unlikely to be the case for H5N1 in dairy cattle given the estimated attack rate on farms — one study found almost 90% of cattle were seropositive following an outbreak (the expected attack rate increases with basic reproduction number). To fully quantify the effect of case isolation and frequent pooled testing on the size of H5N1 outbreaks on dairy farms would require a mathematical model describing the on-farm epidemiological transmission dynamics.”

We have also included additional text in the case isolation results section, to discuss more uncertainty around the effectiveness of case isolation:

“However, it is currently unclear what proportion of naturally infected cattle exhibit identifiable symptoms, if the timing is comparable to experimentally infected cattle, **nor if infectiousness is correlated with the severity (and therefore identifiability) of symptoms**.

Including discussion of a recent paper that suggests a high fraction of cases are subclinical (so unlikely case isolation will be successful as hard to identify high proportion of infections):
“A recent seroprevalence study, following an outbreak of H5N1 on a dairy farm, estimated that approximately 75% of infections were subclinical [13]; although this value will be sensitive to the definition of clinical infection used (i.e. severity/identifiability of the symptoms used to define a clinical case).”
“In general, higher proportions of and faster rates of infection detection will result in greater effectiveness of case isolation. **However, it will be difficult for high proportions of infections to be detected if a high fraction of infections are subclinical, as has recently been estimated** [13].”

Finally, I was intrigued to see the acknowledgement of Steven Moratti about helpful discussions around dairy farming practices. There were a few references to typical dairy practices throughout the paper, and I admit that my curiosity was piqued each time. To the extent that the authors and editor feel it would be appropriate, I would suggest a brief

explanation in the Introduction of typical dairy practices, the route of H5N1 transmission, the natural history of infection, and common interventions. My hope would be that even a few sentences here would help readers to understand the context of the modeling, particularly given the disproportionate focus of the infectious disease modeling community on human hosts.

We have included some brief discussion of relevant dairy practices when we first discuss case isolation:
"...given case isolation (Fig.3, SFig.4) — a standard practice for preventing transmission of other pathogens affecting dairy cattle [12]."
And later when discussing effects of pooled surveillance and case isolation more generally (see above):
"We considered the effectiveness of case isolation and pooled testing of milk samples in isolation but did not consider their combined effect. If pooled testing was conducted regularly, outbreaks on dairy farms could be detected rapidly, allowing for the timely implementation of interventions. **Case isolation is used for preventing within-farm transmission of certain diseases commonly afflicting dairy cattle (e.g. mastitis (Dairy Australia 2018))**. We inferred the effect of case isolation on transmission, estimating the proportion of transmission prevented, as a function of the proportion of infections isolated, and the time from infection to isolation. However, the effect of a transmission reducing intervention on the overall size of an outbreak will be highly dependent on the inherent transmissibility of the pathogen (i.e. the basic reproduction number, R0: the expected number of secondary infections arising from a primary infection in an otherwise susceptible population), and how soon after the onset of the outbreak the intervention is implemented. The earlier interventions are implemented the smaller the outbreak will be. The higher the basic reproduction number (R0>>1) the smaller the impact of interventions will be. Note that if the basic reproduction number is close to one, then only a small reduction in transmission would be required for an outbreak to be controlled (i.e. an immediate reduction in infections). However, this is unlikely to be the case for H5N1 in dairy cattle given the estimated attack rate on farms — one study found almost 90% of cattle were seropositive following an outbreak (the expected attack rate increases with basic reproduction number). To fully quantify the effect of case isolation and frequent pooled testing on the size of H5N1 outbreaks on dairy farms would require a mathematical model describing the on-farm transmission dynamics."

We have additionally commented on the lack of on-farm intervention strategies for H5N1 transmission, so the reader is aware that there aren't necessarily standard practices for dealing with outbreaks of pathogens:
"**This highlights the need for the development of novel on-farm intervention strategies that effectively reduce within-herd transmission.**"
We are currently developing on-farm H5N1 transmission models in order to develop new intervention strategies – this was a large motivation for this work, as we sought estimates for the duration of infectiousness (and other quantities) for parameterising the model.
