# Supplementary material for: Modeling of H5N1 influenza virus kinetics during dairy cattle infection suggests the timing of infectiousness
Source: PLoS Biol. 2026 Jan 5;24(1):e3003586. doi: 10.1371/journal.pbio.3003586 (PMC12782433; doi:10.1371/journal.pbio.3003586)
Supplement: Attachment — Submitted filename: Response to reviewers 2.pdf [file pbio.3003586.s012.pdf]

Dear Editors,

PBIOLOGY-D-25-01949R1

Thank you for your email on 11 December 2025 and your invitation to revise and re-submit our short report previously entitled “Viral kinetics of H5N1 infections in dairy cattle" for further consideration by PLOS Biology.

We thank the editor and reviewers for their helpful comments.

We have addressed all of your comments and addressed reviewer one’s minor comments below. Our responses are in red.

We believe that the revised manuscript has been improved. I would be happy to answer any further questions that you might have and look forward to hearing your decision.

Yours sincerely,

Dr Oliver Eales

Editor's comments:

1) We routinely suggest changes to titles to ensure maximum accessibility for a broad, non-specialist readership, and to ensure they reflect the contents of the paper. In this case, we would suggest a minor edit to the title, as follows. Please ensure you change both the manuscript file and the online submission system, as they need to match for final acceptance:
"Modelling of H5N1 influenza virus kinetics during dairy cattle infection suggests how long animals remain infectious"

We are very happy to change the title of our paper. We suggest: "Modelling of H5N1 influenza virus kinetics during dairy cattle infection suggests the timing of infectiousness", which is very similar to the one you suggested. We would be happy with alternatives though.

2) Please update your abstract to says that you have look to 4 studies, instead of the original 3.
We have updated the abstract accordingly

3) Please add the links of the funding agencies in the Financial Disclosure statement in the manuscript details.

We have added the links as requested.

4) Please cite the location of the data clearly in all relevant main and supplementary Figure legends, e.g. "The data underlying this Figure can be found in S1 Data" or "The data underlying this Figure can be found in https://doi.org/10.5281/zenodo.XXXXX"

We have added a reference to the location of the data at the end of all figures and supplementary figures.

5) Supplementary files (e.g., excel). Please ensure that all data files are uploaded as 'Supporting Information' and are invariably referred to (in the manuscript, figure legends, and the Description field when uploading your files) using the following format verbatim: S1 Data, S2 Data, etc. Multiple panels of a single or even several figures can be included as multiple sheets in one excel file that is saved using exactly the following convention: S1_Data.xlsx (using an underscore).
We have uploaded two files titled S1 Data and S2 data providing the underlying data for all figures and supplementary figures.

Reviewer #1:

This revised submission by Eales et. al. was well responsive to this reviewer's comments. New data was incorporated, in line with newly published studies (increasing robustness). I appreciate the assessment of new parameters (e.g., effects of reduced milk output on pooled testing). Further, the authors delved deeper into potential limitations of their study, which further serve to balance their modeling assumptions with more complex biological associations. This reviewer is satisfied with the manuscript in its current form. However, some very minor points could be addressed.

We thank the reviewer for their supportive comments and all of their helpful feedback throughout the reviewer process.

Minor:
Consider updating the introduction regarding the current state of H5N1 in the U.S.: "As of 21 May 2025, 1070 cases…"
* These aren't truly "cases" as individual testing is not done. Rather, this is "herd-level" surveillance.
* Now in December 2025, the number of positive herds and total number of states affected has changed. Consider updating to reflect the most recent data.

We have updated the text in the introduction accordingly:
"As of **17 November 2025**, **1083 cattle herd outbreaks** have been confirmed across **18** states"

The authors note "…one study that inoculated a single cow with significantly lower volume of virus…" needs correcting. In the report by Lee et. al., four cows were infected, with each cow receiving three different infectious doses that varied by quarter. Please revisit the experimental setup of this study and revise accordingly.

We have updated the text accordingly:
"... that inoculated **four cows** with …"
